# Adolescent Inpatient Mental Health Admissions: An Exploration of Interpersonal Polyvictimization, Family Dysfunction, Self-Harm and Suicidal Behaviours

**DOI:** 10.1007/s10578-022-01450-4

**Published:** 2022-10-31

**Authors:** Shannon L. Stewart, Valbona Semovski, Natalia Lapshina

**Affiliations:** Faculty of Education, The University of Western Ontario, 1137 Western Road, N6G 1G7 London, ON Canada

**Keywords:** interpersonal polyvictimization, trauma, maltreatment, adolescents, inpatient mental health services

## Abstract

The mental health system is impacted by extreme delays in the provision of care, even in the face of suicidal behaviour. The failure to address mental health issues in a timely fashion result in a dependence on acute mental health services. Improvement to the mental health care system is impacted by the paucity of information surrounding client profiles admitted to inpatient settings. Using archival data from 10,865 adolescents 12–18 years of age (*M*_age_ = 14.87, *SD*_age_ = 1.77), this study aimed to examine the characteristics of adolescents admitted to psychiatric inpatient services in Ontario, Canada. Multivariate binary logistic regression revealed that adolescents reporting interpersonal polyvictimization, greater family dysfunction and higher risk of suicide and self-harm had a greater likelihood of an inpatient mental health admission. The interRAI Child and Youth Mental Health assessment can be used for care planning and early intervention to support adolescents and their families before suicide risk is imminent.

## Introduction

Adolescence is a pivotal developmental period of rapid physical, cognitive, social and emotional changes [1–3]. Adolescents are tasked with navigating transitions into certain roles from childhood while acquiring and implementing skills to foster their own well-being and that of subsequent generations [4]. Approximately 75% of mental disorders in adulthood begin before 25 years of age, with onset typically occurring between 11 and 18 years [5]. In Canada, it is estimated that 14% of adolescents suffer from a mental health disorder [6], but approximately 25% of these individuals are receiving professional mental health services [7]. Mental health disorders, especially those of greater severity, negatively influence the adolescent’s overall quality of life. If left untreated, mental health disorders can result in several maladaptive outcomes including increased rates of suicide and hospitalization [6].

At present, suicide is one of the leading causes of death among adolescents and one of the most common reasons for admission to inpatient settings [8]. Traditionally, inpatient admissions were reserved for comprehensive evaluations [9], the stabilization of symptoms and the reduction of imminent risk [10] among individuals with severe behavioural or emotional disturbances [11–13]. However, recent studies have suggested an increase in both emergency room visits and hospitalizations that are not thought to have occurred solely due to medically serious suicide attempts [14] or heightened mental health conditions [15, 16]. The etiology of mental health conditions is complex and is associated with a number of factors, including but not limited to, individual characteristics (e.g., sex) [17, 18], interpersonal relationships [19] and traumatic life events (e.g., physical assault, sexual abuse) [20]. Although reasons for admission to inpatient settings are multifaceted, it is often chosen as the setting of choice because of the prompt care received for mental health needs [21] as families are encountering extreme delays in the provision of care, resulting in the heightened dependence on acute settings [4, 11].

While inpatient settings are instrumental in stabilizing severe mental health disturbances, they may also carry burden [22], such as being costly for the healthcare system [23], having limited capacity [24] and requiring disconnection from family, friends, education and employment. Thus, safe and effective alternatives to inpatient admissions in community settings are favoured [22]. However, advancements in the field are hindered due to the unavailability of data that is collected and reported on service use [16]. In Ontario, Canada, a standardized mental health process has not been yet mandated. This results in inconsistent practices across mental health agencies that negatively impact our understanding of client profiles [25, 26].

Adolescents treated in inpatient settings tend to have several risk factors such as adverse childhood experiences (ACEs), dysfunctional families and increased suicidal behaviours [8]. ACEs are defined as stressful or potentially traumatic events that occur in the first 18 years of an individual’s life and are commonly identified as belonging to distinct categories of trauma (e.g., parental incarceration, violence in the household, emotional abuse/neglect [27]. In contrast to isolated types of ACEs or potentially traumatic life events, the majority of adolescents who have been maltreated will have experienced polyvictimization, which is operationalized here as the exposure to several interpersonal traumatic life events by the same individual across multiple settings [28–31]. Moreover, the concept of polyvictimization captures the interrelationships among trauma types and associated cumulative risk. The present study focuses on interpersonal polyvictimization which refers to the total number of different types of interpersonal traumas experienced by a child or adolescent, and does not characterize a single type of trauma as being more detrimental than another. Moreover, in this study, interpersonal trauma is described as the exposure to physical or sexual assault/abuse and physical, emotional, neglect and unmet safety needs. This conceptualization is different from the definition of ACEs used in the seminal paper by Felitti and colleagues that investigated a range of traumatic events [27]. Recent research has indicated that 61% of children have experienced at least one type of interpersonal trauma and more than a third report two or more types [32]. Due to the relational nature of interpersonal trauma [32], adolescents who have experienced trauma are more vulnerable to higher rates of psychiatric and medical service utilization than peers without traumatic experiences [33]. Much of the research in this area has focused on a single type of ACE [34] and although poly-victimization has been studied in child inpatients, there is limited research geared towards adolescents [35] and interpersonal trauma in particular remains understudied [32]. Thus, the focus of this study is to examine interpersonal polyvictimization and its association with more costly outcomes when compared to non-interpersonal trauma types among adolescents.

Adolescence is also linked to shifts in parent-child relationships with adolescents requiring greater independence. The way in which parents respond to such power struggles has ramifications on the adolescent’s ability to manage emotions and overall well-being which is linked to adolescent psychopathology [2]. The changes in social dynamics during adolescence could widen the authoritative gap, exacerbating the inability for parents and caregivers to manage the adolescent’s behaviours. The inability to manage could result in the family’s reliance on emergency services for aid [36]. Further straining these interactions could be the distress that some families may feel parenting an adolescent with a mental health condition. A recent study found that parents of a child with mental health challenges felt that others were dismissive, misunderstood their child’s disorder and viewed their child negatively [37]. Further, having a family member with a mental health disorder may act as a risk factor for poor family functioning [38]. This weakened family unit could, in turn, negatively affect the youth’s well-being and heighten the possibility for maladaptive outcomes such as hospitalization. However, there is a need for greater research in this area as few studies have investigated the association between family dysfunction and mental health admissions specifically [38].

When investigating sex and age differences in relation to mental health admissions, the results are varied. Studies examining sex differences have been limited. However, a comparable number of females and males are thought to be hospitalized on psychiatric units yearly [39]. An exploration of suicide rates more specifically, however, highlight differences between males and females. Females are more likely to experience emotional or sexual abuse [40, 41], experience higher levels of internalizing symptoms [42–45] and are at greater risk for suicidal ideation and attempts [46] when compared to male counterparts. Thus, females, compared to males, are admitted to inpatient settings more frequently [36, 47]. Still, more research in this area is required that investigates multiple inpatient settings and psychiatric diagnosis [39].

In order to improve timely access to mental health services in the community, high quality assessments utilizing reliable and valid scales and risk algorithms are crucial. Using a large Canadian sample of adolescents, this study aimed to examine the characteristics of those being admitted to inpatient psychiatric services in Ontario to improve our understanding of the factors associated with the use of intensive services. Our study differs from the extant literature by way of using a multimodal approach to data collection, minimizing the reliance on solely retrospective reports and investigating interpersonal polyvictimization rather than selective ACEs. Further, this study investigates the role of family functioning and risk for suicidal ideation/intent related to mental health admissions. Being Canada’s largest province, Ontario’s demographics are considered representative of the country as a whole [39] and is the focus of the present study.

This information is instrumental in improving early intervention and prevention efforts spearheaded by community agencies focused on improving the lives of the most vulnerable adolescents. It is important to address mental health concerns in their infancy before worsening to a point which requires a mental health admission. As such, this information could also be useful in ensuring communities are well equipped for the adolescent’s unique needs upon discharge. Based on the extant literature, it was hypothesized that inpatient admissions would be associated with greater interpersonal polyvictimization, family dysfunction and higher levels of suicidal ideation/intent. It was also anticipated that older age and being female would be associated with a greater likelihood of mental health admissions.

## Materials and Methods

### Procedures

Data were collected from November 2012 to August 2020 by trained assessors at intake into a variety of inpatient or outpatient mental health service agencies located in the Province of Ontario. Trained clinicians (e.g., nurses, social workers, psychologists) conducted a semi-structured interview with the child/adolescent, family members, or other professionals, and also reviewed medical records, report cards, academic assessments, and clinical documentation. Assessors rated the child on a number of demographic variables, family, mental health and physical health indicators. Data was then entered into a de-identified web-based software system using secure storage on the interRAI Canada server. interRAI is a collaborative network of researchers and practitioners in over 35 countries committed to improving the lives of vulnerable persons across the lifespan. Only fully completed assessments could be submitted into the software program. Research Ethics Board approval was granted for secondary analysis of the data collected.

## Participants

The sample included 10,865 adolescents 12–18 years of age (M_age_ = 14.87, SD_age_ = 1.77) who received an initial assessment using the interRAI Child and Youth Mental Health assessment (ChYMH). Adolescents were referred to mental health agencies by their parents, teachers, physicians or other allied healthcare service providers and assessments were conducted as part of standard of care at participating mental health agencies. More than half of the adolescents were females (53.38%; n = 5800). The majority of adolescents in the sample were outpatients (92.95%, n = 10,099), while 7.05% (n = 766) were inpatients. Table 1 presents other relevant demographic characteristics, such as a history of foster family placement, legal guardianship and marital status of parents.

**Table 1:** *Sample characteristics*

	*N*	Percent
**History of foster family placement** ^**1**^		
None	9483	87.22
One foster family	776	7.14
Multiple foster families	614	5.65
**Legal guardianship** ^**2**^		
Both parents	6365	58.45
Mother only	3069	28.18
Father only	504	4.64
Other relatives or non-relatives	483	4.44
Child protection agency	414	3.80
Public guardian	13	0.12
Youth responsible for self	41	0.38
**Marital status of parents** ^**2**^		
Never married	1964	18.05
Married	4512	41.49
Partner/significant other	197	1.81
Widowed	267	2.45
Separated	1328	12.21
Divorced	2013	18.51
Unknown	596	5.48

## Measures

The interRAI Child and Youth Mental Health (ChYMH) [48] assessment is a comprehensive standardized mental health instrument for children and adolescents aged 4 to 18 years. The instrument collects information on a variety of domains (e.g., communication, mental health state indicators, family and social relations and behaviour) for children and adolescents. Information is gathered by trained assessors, who have at least two years of clinical experience working with children or adolescents. Assessors received a two-day training program focused on the administration of the instrument and used direct contact with the family, child, other service providers and any collateral information to complete the instrument. The interRAI ChYMH is part of an integrated health information assessment system and has demonstrated strong reliability and validity [26, 48–53].

## Mental Health Admissions

Mental health residential/inpatient history was assessed utilizing an item related to the number of lifetime admissions, with response options 0 – none, 1– 1 to 3, 2 –4 to 5, 3– 6 or more. Due to small counts of any admissions (coded 1–3), responses were further dichotomized into 0- none and 1- any lifetime admission.

## Interpersonal Trauma

Interpersonal traumatic life events were assessed with two items: being a victim of physical or sexual assault or abuse. Item responses ranged from 0- never, 1- more than 1 year ago, 2– 31 days to 1 year ago, 3– 8 to 30 days ago, 4– 4 to 7 days ago, and 5- present within the last 3 days. In addition, three questions assessed neglect: physical, emotional neglect, and unmet safety needs, with responses ranging from 0- none, 1 − 0 to 4 years, 2– 5 to 11 years, 3– 12 to 18 years, indicating the child’s age at the earliest occurrence. Due to the low prevalence of recent interpersonal traumatic life events, responses were dichotomized into 0 – never/none and 1 – more than one year ago to in the last three days/0 to 4 years-12 to 18 years. Next, the three neglect items were combined into one item which indicated the presence of neglect (0 – none, 1 – yes). The number of interpersonal traumas present were then summed to create an ordinal interpersonal polyvictimization variable, with values of 0 (no trauma), 1 (one type of trauma), 2 (two types of trauma), and 3 (three types of trauma).

## Family Functioning

Five items assessed family functioning: strong and supportive relationship with family, family are persistently hostile or critical of child, parent has mental health issues, sibling has mental health issues, parent is unwilling or unable to continue care for child. Response options included 0 – no, 1 – yes, 8 – unknown or not applicable. Cases with responses coded as “8” were excluded from the sample and analyses. Items were recoded where necessary. Responses were combined into a single ordinal variable with values ranging from 0 to 5, where higher scores indicated higher degree of family dysfunction.

## Risk of Suicide and Self-Harm in Kids (RiSsK)

Risk for harm to self was measured using the Risk of Suicide and Self-Harm in Kids (RiSsK) algorithm, which reflects the risk of suicide and self-harm among children and adolescents [51]. The RiSsK algorithm decision tree is composed of six individual items (i.e., attempt to kill, self-harm attempt without intent to kill, considered self-injury, others concerned about self-injury, family overwhelmed, and any self-injurious behaviours) from the ChYMH assessment, as well as the Depression Severity Index (DSI; a nine-item measure of the frequency and severity of depressive symptoms). The RiSsK decision tree is composed of twenty terminal nodes ranging from zero to six, where higher risk levels are indicative of greater risk for suicide and self-harm. Validation research indicated that the RiSsK algorithm has strong psychometric properties and clinical applicability among clinically referred children and adolescents for indicating risk of suicide and self-harm. Due to the potential for associated life-threatening outcomes, researchers argued the necessity for a high severity risk cut-point that favoured sensitivity over specificity. A cut-point of 2 + was determined to provide adequate sensitivity (93%) and specificity (61%) indicative of risk of suicide and self-harm among a clinical population of children and adolescents [51].

## Analytic Strategy

The results were analyzed using SAS software package, version 9.4. Frequency analyses were conducted to examine demographic variables. Pearson chi-square analyses were utilized to examine the relationships among categorical predictors (sex and interpersonal polyvictimization) and the outcome. Univariate binary logistic regression models assessed the relationships between interval predictors (age, RiSsK, family functioning) and the outcome variable. To test the hypothesis, a series of hierarchical multivariate binary logistic regression models were compared. Model 1 included only the demographic predictors (sex and age). In Model 2, RiSsK was added to sex and age. Finally, Model 3 included sex (male, female), age, RiSsK, interpersonal polyvictimization and family functioning as covariates. The dependent variable was lifetime mental health admissions (yes, no). In the model, no trauma and female served as reference categories for interpersonal polyvictimization and sex respectively. All statistical tests were two-tailed. The significance level was set at alpha 0.05, which corresponded to 95% confidence intervals in binary logistic regression analyses.

## Results

### Preliminary Results

#### Relationships Among Predictor Variables and Mental Health Admissions

Pearson’s chi-square analyses were conducted to examine the relationships. Table 2 provides frequencies of sex and interpersonal polyvictimization related to mental health admissions in the sample. More females than males experienced lifetime mental health admissions. Adolescents with a higher number of traumatic life events were more likely to be admitted to mental health services.

**Table 2:** *Descriptive statistics of categorical predictor variables as a function of mental health admissions (N = 10,867)*

Predictor variable		No admissions percent	Admissions percent	*X* ^*2*^	*p*	*Phi/Cramer’s v*
Sex	Male	77.36	22.64	63.97	< 0.0001	0.077
	Female	70.56	29.41			
Interpersonal Polyvictimization^1^	No trauma	78.75	21.25	346.55	< 0.0001	0.179
	One trauma	68.88	31.12			
	Two traumas	61.39	38.61			
	Three traumas	45.66	54.34			

*Notes*. ^1^* N* = 10,863

Next, we examined the relationships among ordinal/interval predictors and mental health admissions. As seen in Table 3, compared to adolescents with no mental health admission history, adolescents who experienced any mental health admission were older, scored higher on RiSsK and family dysfunction.

**Table 3:** *Univariate binary logistic regression models for mental health admissions as a function of predictor variables*

Predictor variable	OR	95% OR CI	*Wald X* ^*2*^	*p*	*Max-rescaled R* ^*2*^
Age	1.21	1.18, 1.24	218.24	< 0.0001	0.03
RiSsK	1.62	1.58, 1.67	1150.13	< 0.0001	0.17
Family functioning	1.31	1.26, 1.36	198.12	< 0.0001	0.03

*Notes*. Age range: 12–18 years; RiSsK = Risk of Suicide and Self-Harm in Kids (range: 0–6, where higher scores indicate greater risk); Family functioning range: 0–5, where higher scores indicate greater family dysfunction

## Primary Results

To test the hypothesis, multivariate binary logistic regression was utilized, where the lifetime inpatient mental health admission variable was regressed onto RiSsK, interpersonal polyvictimization, family functioning, sex and age. Table 4 provides odds ratios, 95% confidence intervals, and statistical significance for predictors in the three models, as well as the model fit.

Model 1 included sex (male vs. female) and age as predictors. In this model, males were less likely in odds to be admitted to mental health services. Older age was associated with the greater likelihood in odds of lifetime admission.

Model 2 included sex, age, and RiSsK score as predictors. This model demonstrated better fit than Model 1. In this model, the relationship between sex and inpatient mental health admissions reversed compared to Model 1. Older adolescents were more likely in odds to be admitted, as well as those with higher risk of self-harm.

Model 3 included all predictors. Controlling for other predictors in the model, male sex (vs. female) and older age were related to a greater likelihood of inpatient mental health admissions. In addition, higher RiSsK scores and greater family dysfunction were related to a greater likelihood in odds of mental health admissions (vs. no admissions). Compared to adolescents with no trauma, those with one, two, or three traumas were more likely in odds to experience mental health admission; this likelihood increased as the number of traumas increased.

**Table 4:** *Hierarchical binary logistic regression analyses of mental health admissions as a function of predictor variables (N = 10,867)*

		Model 1		Model 2		Model 3^1^	
**Predictor**		OR (95% CI)	*p*	OR (95% CI)	*p*	OR (95% CI)	*p*
Sex (male)		0.76 (0.69, 0.82)	< 0.0001	1.16 (1.05, 1.28)	0.0031	1.19 (1.08, 1.31)	0.0006
Age		1.20 (1.17, 1.23)	< 0.0001	1.16 (1.13, 1.19)	< 0.0001	1.15 (1.12, 1.18)	< 0.0001
RiSsK				1.62 (1.60, 1.66)	< 0.0001	1.58 (1.53, 1.63)	< 0.0001
Interpersonal Polyvictimization							
	One trauma type					1.36 (1.22, 1.53)	< 0.0001
	Two trauma types					1.60 (1.38, 1.86)	< 0.0001
	Three trauma types					2.64 (2.07, 3.36)	< 0.0001
Family functioning						1.13 (1.08, 1.17)	< 0.0001
Max-scaled *R*^2^		0.04		0.18		0.20	
Likelihood ratio *X*^2^		263.86	< 0.0001	1427.21	< 0.0001	1596.32	< 0.0001

*Notes*: ^1^* N* = 10,863. Age range: 12–18 years; RiSsK = Risk of Suicide and Self-Harm in Kids (range: 0–6, where higher scores indicate greater risk); Family functioning range: 0–5, where higher scores indicate greater family dysfunction

## Discussion

Using a sample of Canadian adolescents, the current study examined specific factors associated with lifetime inpatient mental health admissions in this population. To our knowledge, this is one of the few recent Canadian studies commenting on specific characteristics that increase vulnerability to acute mental health services for adolescents. As hypothesized, adolescents exposed to interpersonal polyvictimization reported greater family dysfunction, exhibited greater suicidal ideation/intent, and were more likely in odds to have experienced admission to inpatient mental health services compared to adolescents without such exposure. These findings are consistent with evidence in the literature. Below we discuss the main study findings.

## Interpersonal Polyvictimization

The present study assessed among other factors, associations among traumatic exposures to physical abuse, sexual assault or abuse, or neglect and the likelihood of inpatient mental health admissions in a sample of clinically-referred adolescents. Adolescents with a higher number of interpersonal traumatic life events (one, two, or three traumas) were more likely in odds to be admitted to inpatient mental health services compared to those who did not experience these types of trauma, further supporting findings that maltreatment may be associated with a higher need for more urgent and immediate services. Furthermore, our finding also supports that adolescents who have experienced maltreatment of an interpersonal nature (e.g., emotional abuse, sexual assault) are more likely to utilize psychiatric and medical services than adolescents without such experiences [33, 54]. Marshall et al. [55] found that adolescents who reported interpersonal trauma were 65% more likely to require urgent mental health services than peers who did not report interpersonal trauma. Additionally, adolescents who have experienced maltreatment are at an increased risk of being exposed to other forms of adversity [56]. For example, these adolescents may live in environments marked with heightened distress (e.g., family dysfunction characterized by parenting behaviours that may be detrimental to adolescent development especially, in the absence of supports such as respite care) [57].

## Family Functioning

The risk for mental disorders is associated with an adolescents’ social context [58]. In this study, controlling for other predictors, for each point increase in impaired family functioning we found that adolescents were more likely in odds to be admitted for mental health inpatient services. Family relationships are arguably one of the most important social relationships experienced in childhood, as they are among the first to set the stage for the child’s physical, psychological and emotional growth [59]. In adolescence, children are simultaneously requiring greater independence and distance from their caregivers, potentially causing some conflict within the household. The way in which caregivers respond to these changes has ramifications on the adolescent’s response to emotional stimuli. For example, better communication has been associated with greater self-esteem, social functioning and fewer mental health issues [2].

Contrarily, adolescents who are faced with unsupportive familial relationships [60], hostility in the home [61] or may have a parent [62–64] or sibling with mental health issues [64] may be at greater risk for their own mental health concerns. Adolescents living in such environments are at an increased risk of suicide, substance use, mood disorders and issues with eating [2]. To explain the linkage between chronic interpersonal stressors and the presentation of mental health disorders is the stress generation model [19]. In the context of depression, the stress generation model suggests that adolescents who are prone to depression, compared to those without a vulnerability to internalizing issues, in some way shape outcomes related to interpersonal conflict due to personal characteristics (e.g., cognitive style) and maladaptive behaviours. This places them at higher risk for subsequent depression and/or the maintenance of current depressive episodes [65]. This theory has been extended to anxiety and externalizing issues as well [66]. In the absence of healthy coping mechanisms, adolescents who are faced with chronic interpersonal stressors may require support via acute mental health settings.

## Suicide and Self-Harm

In our study, controlling for other predictors, for each point increase in the RiSsK score, an adolescent was 58% more likely in odds to be admitted for mental health services. Self-harm and suicidality are among one of the most common reasons for mental health inpatient admissions [8, 13, 67]. Children admitted to acute mental health services for self-harm and suicidal behaviours are believed to have greater mental health issues when compared to peers admitted for other reasons [68]. These adolescents also possess more risk factors heightening their vulnerability including low self-esteem, maltreatment and dysfunctional families [8]. Studies have shown that more than half of adolescents who are suicidal report family dysfunction predating their suicidal behaviours [69]. Poor relationships with caregivers may exacerbate emotion dysregulation and result in maladaptive coping mechanisms [70], further underscoring the importance of considering these factors together when working with this population. For some families, it is possible that adolescents at heightened risk for suicide may also cause significant family distress due to increased need for monitoring and concerns regarding their emotional state.

This connection is important to understand and investigate as trends of mental health visits related to self-harm in Ontario have increased in recent years [71], especially in the adolescent population [72]. Aside from the increased cost associated with inpatient services, inpatient settings have been criticized as being inappropriate for long-term alleviation of symptoms [23] and for the treatment of self-harming behaviours [67]. This is problematic as previous suicide attempts act as a risk factor for completed suicide following an inpatient mental health admission. Additionally, in the month following a mental health inpatient admission, it is believed that the suicide death rate is much higher for those admitted when compared to the general population [73, 74]. Advancing our knowledge on suicidal behaviour among inpatients can aid in creating more effective, efficient and appropriate treatments as well as standardized measures using high-quality data to evaluate intervention efforts [8].

## Age and Sex Differences

Although not a main focus in this study, taking into account other predictors, older age and being a male was associated with a greater likelihood in odds of mental health admissions. In relation to age, this finding was in line with our hypothesis. Generally, adolescents compared to preadolescents, also have increased opportunities to engage in risky behaviours (e.g., physical aggression, running away) and are at greater risk of expressing symptoms related to serious emotional or personality disorders that would prompt urgent intervention [75]. Furthermore, there are sex differences highlighted in the literature related to suicidal behaviour [76]. An observation in the present study that warrants attention and underscores the assertion that there are sex/gender differences in relation to suicidal behaviours is the reversed effect of sex between Model 1 and Models 2 and 3. In Model 1, as predicted, females were more likely in odds to be admitted to inpatient mental health services. However, contrary to our prediction, in Model 2, males were more likely in odds to receive inpatient admission once the risk of suicide and self-harm was accounted for in the regression analysis. This pattern remained in Model 3, wherein males with the same level of RiSsK, interpersonal trauma and family dysfunction as females were more likely in odds to be admitted to inpatient mental health services.

The finding that, controlling for other predictors, the addition of the risk of suicidality and self-harm reverses the relationship between sex and inpatient mental health admissions may be explained by the “gender paradox” [77]. Namely, it suggests that suicide attempts and suicidal thoughts are greater in females, but suicide death rates are greater in males. Additionally, this gender paradox may be further explained by the lethality of methods chosen by males intending to commit suicide. Boyd et al. [78] found that females who are disproportionately impacted by internalizing disorders may result in higher rates of suicide attempts whereas externalizing disorders in males could result in the higher death rates by suicide. Our study’s findings also suggest that the mental health care sectors’ response to suicidal behaviours differs between the sexes. The literature has suggested that females are required to express greater impairment than males for similar mental health difficulties to warrant a referral and subsequently, access to mental health services [79]. This finding may encompass suicidality as well, as our study would lend support for this assertion. The differences between male and female adolescents may result in males having a greater likelihood in odds of being admitted to inpatient mental health services over females, despite similar clinical profiles.

## Implications for Practice

For some adolescents, inpatient mental health services are necessary to treat their mental health concerns. Admittance to such services may introduce difficulties for adolescents because they are separated from their social networks, school and employment. Thus, inpatient mental health services are designed to stabilize immediate risk and should prepare individuals for life following admission. Less intensive services should be utilized whenever possible to circumvent the reliance of acute, intensive resources for adolescents who can benefit from outpatient settings. Recent research findings suggest that mental health services geared towards adolescents are not meeting their unique needs [80] indicating a need to develop risk adjusted, quality indicators designed for children and youth in both inpatient and outpatient services. Poor discharge practices perpetuate fragmented care coordination, heightened risk of relapse [81], and inadequate access to services resulting in poorer clinical outcomes and a reliance on acute mental health care settings [82].

Thus, community services need to be strengthened to better suit the needs of the most vulnerable adolescents, especially considering many of the presenting issues can be effectively treated in communities [12]. In strengthening community services, especially upon discharge from inpatient settings, it is important to recognize the factors that are associated with inpatient admissions, re-hospitalization risk and the best practices in care transition. This information can assist clinicians in adequately addressing difficulties experienced by adolescents and potentially prevent future hospitalizations. Findings reported herein indicated that interpersonal polyvictimization, family functioning and risk of suicide and self-harm were associated with inpatient mental health admissions. This finding emphasizes the need for strong family and social support systems given that poor family functioning and maltreatment is a foreboding prognostic factor [83]. To foster improved treatment sustainability and potentially prevent future re-admissions, adopting a trauma-informed lens that addresses maltreatment, family difficulties and suicide risk levels is needed [84]. Improved family functioning, in particular, has been linked to maintenance of improvements upon discharge from an inpatient setting [23]. Given that adolescents who experience maltreatment are at heightened risk of engaging in suicidal behaviours [57], addressing family violence, emotional abuse, and exposure to other trauma-related experiences can reduce the risk of suicide and connect adolescents to adequate care for their needs.

To guide the decision making for mental health care professionals, The National Action Alliance for Suicide Prevention [74] has outlined best practices in care transitions for individuals with suicide risk. Upon discharge from an inpatient setting, there are several evidence-based recommendations provided that underscore some of the aforementioned factors highlighted in our study. The most salient theme permeating the guidelines is the importance of a thorough assessment and prompt triaging to services based on the adolescent’s specific needs. This is not surprising, considering that timely referrals to community agencies after hospitalization are critical to suicide prevention [84]. Hindering the smooth and prompt transition between services is the lack of coordination between service sectors [4, 85] and the absence of standardized processes for data collection and sharing across the province [25]. A new standardized assessment-to-intervention system spanning multiple service sectors, provides best practice care planning protocols to reduce risks associated with re-admission. This is possible by bolstering support systems after discharge, and evaluating outcomes while addressing resource allocation. This has been designed and utilized across multiple countries to support service system integration (www.interRAI.org) [50, 86]. This well-established approach utilizes high-quality data from across the lifespan (from infancy to elder care) to support improved care for vulnerable populations and has been recommended by The Child and Youth Mental Health Lead Agency Consortium for use across a variety of service sectors [87] and is utilized across multiple provinces and countries.

A common assessment system that utilizes psychometrically sound algorithms to identify adolescents at-risk is the first step in designing an integrated approach across service sectors [25, 88]. The use of a common standardized tool, such as the interRAI suite of instruments, would provide agencies a common language to communicate and facilitate smooth transitions. Such instruments can also assist with decision making as they have extensively been studied and used internationally. They support improved identification of at-risk adolescents through the collection of high-quality data and through best practice initiatives (e.g., creating collaborative safety plans, involving family members, other caregivers and informal supports). More specifically, the interRAI tools designed for children and youth contain best practice and evidence-informed collaborative action plans (i.e., transitions and support systems for discharge) that target the issues highlighted in our study (i.e., risk of suicide and self-harm) [89]. Alongside the collaborative action plans are algorithms that can inform the mental health professional about the adolescent’s need for urgent and emergent services when seen in the community (i.e., interRAI Children’s Algorithm for Mental Health and Psychiatric Services; ChAMhPs) [90]. The large data bases generated by the interRAI Child and Youth system will also provide a unique opportunity to develop risk-adjusted performance indicators to facilitate quality care and guidelines for agencies and organizations.

The poor transition between mental health service sectors and the varied practices within mental health services for adolescents is not unique to Canada, so we are confident that the implications for clinical care outlined can be incorporated internationally. Inpatient settings in Ontario are said to resemble settings globally in terms of their characteristics (e.g., size of settings, number of beds, services offered) and the prevalence of suicidal behaviours [13]. A stronger understanding of the process that contributed to an adolescent’s inpatient admission is important for treatment and instrumental in strengthening community services. Community services provide the possibility to treat a larger number of adolescents with mental health struggles and, in turn, potentially reduces the number of patients voluntarily seeking services in acute mental health settings [8, 13] and those being involuntarily admitted [13]. Finally, the adoption of interRAI tools locally and abroad can enhance the provision of mental health services and create a strong, effective and integrated network of healthcare professionals dedicated to improving the lives of adolescents.

## Limitations and Future Research

Although the large Canadian sample and use of a reliable, valid and standardized assessment for data collection serve as strengths of the present study, there are notable limitations. First, the study design was cross-sectional. Cross-sectional designs hinder our ability to comment on causal relationships between interpersonal polyvictimization, family functioning and suicidal ideation/intent and mental health admissions. Longitudinal research is currently underway. Secondly, due to the nature of data collection, the study is vulnerable to underreporting of abuse and neglect. To circumvent this issue, multiple sources of information (e.g., assessment reports, school records, child welfare information) were utilized to obtain a comprehensive understanding of the adolescents and the context in which they are embedded. Due to the low number of recent cases of each interpersonal traumatic life event (e.g., physical assault, sexual abuse), these variables were first dichotomized and then combined into a single variable. The nature of this variable precludes the ability to comment on the unique associations of each respective interpersonal trauma type and mental health admissions. Furthermore, the chronicity and severity of the experienced interpersonal traumas were not captured in this study. Chronicity and severity of maltreatment can increase the risk for serious mental health issues [91]. Thus, future research should explore the effects of each interpersonal traumatic life event while including information on the chronicity and severity of the experience. Thirdly, demographic variables such as the adolescent’s socioeconomic status, race and ethnicity were not reported in this study. Future research should also investigate the sex and age differences that were identified in this study alongside the demographic variables. Previous research has identified differing mental health presentations based on a child’s age [17] and between sexes [92]. Results from future studies would inform prevention and treatment services that are specific to the adolescent’s needs based on their sex and age. Future research should explore the variables included and those omitted from the present study using experimental designs to investigate causal relationships.

## Summary

In summary, in addition to male sex and older age, greater interpersonal polyvictimization, greater family dysfunction and higher risk of suicide and self-harm were associated with inpatient mental health admissions. Adolescents will inevitably find themselves accessing acute services for their needs due to the lack of pathways for appropriate and timely care in their communities. Once adolescents are discharged from hospital, strong efforts need to be made to curate a discharge plan that suit their specific needs and connect them to appropriate community resources. This will aid in the maintenance of improvements to their mental health and reduce the likelihood of the need for future inpatient mental health services. Once connected to community resources, agencies should strive to adopt a trauma-informed lens when working with these adolescents, conduct suicide risk assessments as necessary, and engage families, wherever possible, to ensure favourable outcomes for this group of young people.

## References

[1] Lu W, Todhunter-Reid A, Mitsdarffer ML, Muñoz-Laboy M, Yoon AS, Xu L (2021). Barriers and facilitators for mental health service use among racial/ethnic minority adolescents: A systematic review of literature. Front Public Health.

[2] Patton GC, Sawyer SM, Santelli JS, Ross DA, Afifi R, Allen NB (2016). Our future: a Lancet commission on adolescent health and wellbeing. The Lancet.

[3] Vyas NS, Birchwood M, Singh SP (2015). Youth services: meeting the mental health needs of adolescents. Ir J Psychol Med.

[4] Malla A, Shah J, Iyer S, Boksa P, Joober R, Andersson N (2018). Youth mental health should be a top priority for health care in Canada. Can J Psychiatry.

[5] Kessler RC, Berglund P, Demler O, Jin R, Merikangas KR, Walters EE (2005). Lifetime prevalence and age-of-onset distributions of DSM-IV disorders in the National Comorbidity Survey Replication. Arch Gen Psychiatry.

[6] Alimi IO, Mathies I, Archibald A, Compton C, Keku E (2021). Improving child mental health policy in Canada. Cureus.

[7] Schwean V, Rodger S (2013). Children first: It’s time to change! Mental health promotion, prevention, and treatment informed by public health, and resiliency approaches. Can J Sch Psychol.

[8] Kronström K, Tiiri E, Jokiranta-Olkoniemi E, Kaljonen A, Sourander A (2019). Suicidality among child and adolescent psychiatric inpatients: time trend study comparing 2000 and 2011. Eur Child Adolesc Psychiatry.

[9] Zambrowicz R, Stewart JG, Cosby E, Esposito EC, Pridgen B, Auerbach RP (2019). Inpatient psychiatric care outcomes for adolescents: A test of clinical and psychosocial moderators. Evidence-Based Practice in Child and. Adolesc Mental Health.

[10] Lamb CE (2009). Alternatives to admission for children and adolescents: providing intensive mental healthcare services at home and in communities: what works?. Curr Opin Psychiatry.

[11] Hayes C, Simmons M, Palmer VJ, Hamilton B, Simons C, Hopwood M (2020). A profile of adolescents admitted to a private inpatient unit and mental health outcomes. Australas Psychiatry.

[12] Coates D (2018). Service models for urgent and emergency psychiatric care: An overview. J Psychosoc Nurs and Ment Health Serv.

[13] Greenham SL, Persi J (2014). The state of inpatient psychiatry for youth in Ontario: Results of the ONCAIPS Benchmarking Survey. J Can Acad Child Adolesc Psychiatry.

[14] Roberts N, Booij L, Axas N, Repetti L (2016) Two-year prospective study of characteristics and outcome of adolescents referred to an adolescent urgent psychiatric clinic. Int J Adolesc Med Health 30(1). /j/ijamh.2018.30.issue-1/ijamh-2016-0006/ijamh-2016-0006.xml 10.1515/ijamh-2016-0006 10.1515/ijamh-2016-0006 27176739

[15] Clisu DA, Layther I, Dover D, Viner RM, Read T, Cheesman D, Hodges S, Hudson LD (2022). Alternatives to mental health admissions for children and adolescents experiencing mental health crises: A systematic review of the literature. Clin Child Psychol Psychiatry.

[16] Gandhi S, Chiu M, Lam K, Cairney JC, Guttmann A, Kurdyak P (2016). Mental health service use among children and youth in Ontario: Population-based trends over time. J Can Acad Child Adolesc Psychiatry.

[17] Kessler RC, Amminger GP, Aguilar-Gaxiola S, Alonso J, Lee S, Üstün TB (2007). Age of onset of mental disorders: A review of recent literature. Curr Opin in Psychiatry.

[18] Riecher-Rössler A (2017). Sex and gender differences in mental disorders. Lancet Psychiatry.

[19] Cohen JR, Spiro CN, Young JF, Gibb BE, Hankin BL, Abela JRZ (2015). Interpersonal risk profiles for youth depression: A person-centered, multi-wave, longitudinal study. J Abnorm Child Psychol.

[20] McLaughlin KA, Lambert HK (2017). Child trauma exposure and psychopathology: Mechanisms of risk and resilience. Curr Opin in Psychol.

[21] Edwards D, Evans N, Gillen E, Longo M, Pryjmachuk S, Trainor G, Hannigan B (2015). What do we know about the risks for young people moving into, through and out of inpatient mental health care? Findings from an evidence synthesis. Child Adolesc Ment Health.

[22] Clisu DA, Layther I, Dover D, Viner RM, Read T, Cheesman D, Hodges S, Hudson LD (2022). Alternatives to mental health admissions for children and adolescents experiencing mental health crises: A systematic review of the literature. Clin Child Psychol Psychiatry.

[23] Green J, Jacobs B, Beecham J, Dunn G, Kroll L, Tobias C (2007). Inpatient treatment in child and adolescent psychiatry–a prospective study of health gain and costs. J Child Psychol Psychiatry.

[24] O’Herlihy A, Worrall A, Lelliott P, Jaffa T, Hill P, Banerjee S (2003). Distribution and characteristics of in-patient child and adolescent mental health services in England and Wales. Br J Psychiatry.

[25] Duncan L, Boyle MH, Abelson J, Waddell C (2018). Measuring children’s mental health in Ontario: policy issues and prospects for change. J Can Acad Child Adolesc Psychiatry.

[26] Stewart SL, Hirdes JP (2015) Identifying mental health symptoms in children and youth in residential and in-patient care settings. In Healthcare management forum; SAGE Publications: Los Angeles, United States of America; Volume 28, Number 4, pp. 150–156 10.1177/0840470415581240 26015486

[27] Felitti VJ, Anda RF, Nordenberg D, Williamson DF, Spitz AM, Edwards V (1998). Relationship of childhood abuse and household dysfunction to many of the leading causes of death in adults. The Adverse Childhood Experiences (ACE) Study. Am J Prev Med.

[28] Babchishin LK, Romano E (2014). Evaluating the frequency, co-occurrence, and psychosocial correlates of childhood multiple victimization. Can J Commun Ment Health.

[29] Feng J, Hsieh Y, Hwa H, Huang C, Wei H, Shen AC (2019). Childhood poly-victimization and children’s health: A nationally representative study. Child Abuse Negl.

[30] Finkelhor D, Ormrod RK, Turner HA (2007). Poly-victimization: A neglected component in child victimization. Child Abuse Negl.

[31] Julian D, Ford JD, Brianna C, Delker BC (2018). Polyvictimization in childhood and its adverse impacts across the lifespan: Introduction to the special issue. J Trauma Dissociation.

[32] Dugal C, Bigras N, Godbout N, ClaudeBélanger C (2016) Childhood interpersonal trauma and its repercussions in adulthood: An analysis of psychological and interpersonal sequelae. In G. El-Baalbaki, & C. Fortin (Eds.), A multidimensional approach to post-traumatic stress disorder - from theory to practice. IntechOpen. 10.5772/64476

[33] De Bellis MD (2001). Developmental traumatology: The psychobiological development of maltreated children and its implications for research, treatment, and policy. Dev Psychopathol.

[34] Grasso DJ, Dierkhising CB, Branson CE, Ford JD, Lee R (2016). Developmental patterns of adverse childhood experiences and current symptoms and impairment in youth referred for trauma-specific services. J Abnorm Child Psychol.

[35] Ford JD, Elhai JD, Connor DF, Frueh BC (2010). Poly-victimization and risk of posttraumatic, depressive, and substance use disorders and involvement in delinquency in a national sample of adolescents. J Adolesc Health.

[36] Amianto F, Arletti L, Baietto C, Davico C, Migliaretti G, Vitiello B (2021). Trends in admissions to a child and adolescent neuropsychiatric inpatient unit in the 2007–2017 decade: how contemporary neuropsychiatry is changing in Northwestern Italy. Eur Child Adolesc.

[37] McKeague L, Hennessy E, O’Driscoll-Lawrie C, Heary C (2022). Parenting an adolescent who is using a mental health service: A qualitative study on perceptions and management of stigma. J Fam Issues.

[38] Tan J, Conlon C, Tsamparli A, O’Neill D, Adamis D (2019) The association between family dysfunction and admission to an acute mental health inpatient unit: a prospective study. Ir J Psychol Med 1–11 Advance online publication. https://doi-org.proxy1.lib.uwo.ca/10.1017/ipm.2019.41 10.1017/ipm.2019.41 31511120

[39] Vigod SN, Kurdyak P, Fung K, Gruneir A, Herrmann N, Hussain-Shamsy N (2016). Psychiatric hospitalizations: A comparison by gender, sociodemographics, clinical profile, and postdischarge outcomes. Psychiatric Serv.

[40] Moody G, Cannings-John R, Hood K, Kemp A, Robling M (2018). Establishing the international prevalence of self-reported child maltreatment: a systematic review by maltreatment type and gender. BMC Public Health.

[41] Nolen-Hoeksema S (2001). Gender differences in depression. Curr Dir Psychol Sci.

[42] Achenbach TM, Ivanova MY, Rescorla LA, Turner LV, Althoff RR (2016). Internalizing/externalizing problems: Review and recommendations for clinical and research applications. J Am Acad Child Adolesc Psychiatry.

[43] Asscher JJ, Van der Put CE, Stams GJJM (2015). Gender differences in the impact of abuse and neglect victimization on adolescent offending behavior. J Fam Violence.

[44] Stewart S, Toohey A, Lapshina N (2020). Childhood maltreatment and risk of harm to self and others: The role of sex and polyvictimization. Int J Child Adolesc Resil.

[45] Vaillancourt T, Haltigan JD, Smith I, Zwaigenbaum L, Szatmari P, Fombonne E (2017). Joint trajectories of internalizing and externalizing problems in preschool children with autism spectrum disorder. Dev Psychopathol.

[46] Soler L, Segura A, Kirchner T, Forns M (2013). Polyvictimization and Risk for Suicidal Phenomena in a Community Sample of Spanish Adolescents. Violence Vict.

[47] Skinner R, McFaull S, Draca J (2016). Suicide and self-inflicted injury hospitalizations in Canada (1979 to 2014/15). HPCDP.

[48] Stewart SL, Hirdes JP, Curtin-Telegdi N, Perlman C, MacLeod K, Ninan A (2015). interRAI Child and Youth Mental Health (ChYMH) Assessment Form and User’s Manual. Version 9.3.

[49] Stewart SL, Hirdes JP, Curtin-Telegdi N, Perlman C, MacLeod K, Ninan A (2015). interRAI Child and Youth Mental Health (ChYMH) Assessment Form and User’s Manual. Version 9.3.

[50] Hirdes JP, van Everdingen C, Ferris J, Franco-Martin M, Fries BE, Heikkilä J (2020). The interRAI suite of mental health assessment instruments: An integrated system for the continuum of care. Front Psychiatry.

[51] Stewart SL, Celebre A, Hirdes JP, Poss JW(2020) Risk of Suicide and Self-harm in Kids: The development of an algorithm to identify high-risk individuals within the children’s mental health system.Child Psych Human Dev1–12 10.1007/s10578-020-00968-9 PMC7554002 32076912

[52] Stewart SL, Hamza CA (2017). The Child and Youth Mental Health Assessment (ChYMH): An examination of the psychometric properties of an integrated assessment developed for clinically referred children and youth. BMC Health Serv Res.

[53] Stewart SL, Poss JW, Thornley E, Hirdes JP (2019). Resource intensity for children and youth: The development of an algorithm to identify high service users in Children’s Mental Health. Health Serv Insights.

[54] Stewart SL, Leschied A, den Dunnen W, Zalmanowitz S, Baiden P (2013). Treating mental health disorders for children in child welfare care: Evaluating the outcome literature. Child Youth Care Forum.

[55] Marshall C, Semovski V, Stewart SL (2020). Exposure to childhood interpersonal trauma and mental health service urgency. Child Abuse Negl.

[56] Racine N, Dimitropoulos G, Hartwick C, Eirich R, van Roessel L, Madigan S (2021). Characteristics and service needs of maltreated children referred for mental health services at a child advocacy centre in Canada. J Can Acad of Child Adolesc Psychiatry.

[57] Baiden P, Stewart SL, Fallon B (2017) The role of adverse childhood experiences as determinants of non-suicidal self-injury among children and adolescents referred to community and inpatient mental health settings, vol 69. Child Abuse & Neglect, pp 163–176 10.1016/j.chiabu.2017.04.011 28477476

[58] Borschmann R, Patton GC (2018). The outcomes of adolescent mental disorders. Acta psychiatrica Scandinavica.

[59] Berg N, Kiviruusu O, Karvonen S, Rahkonen O, Huurre T(2017) Pathways from problems in adolescent family relationships to midlife mental health via early adulthood disadvantages – a 26-year longitudinal study.PLoS One12(5) 10.1371/journal.pone.0178136 PMC5446125 28552985

[60] Alm S, Brolin Låftman S, Bohman H (2019). Poor family relationships in adolescence and the risk of premature death: Findings from the Stockholm Birth Cohort Study. Int J Environ Res Public Health.

[61] Steinberg L(2000) Youth violence: Do parents and families make a difference? National Institute of Justice Journal (243),31–38

[62] Behere AP, Basnet P, Campbell P (2017). Effects of family structure on mental health of children: A preliminary study. Indian J Psychol Med.

[63] Eun JD, Paksarian D, He J, Merikangas KR (2018). Parenting style and mental disorders in a nationally representative sample of US adolescents. Social Psychiatry and Psychiatric Epidemiology. Int J Res Social Genetic Epidemiol Mental Health Serv.

[64] Ma N, Roberts R, Winefield H, Furber G (2015). The prevalence of psychopathology in siblings of children with mental health problems: A 20-year systematic review. Child Psych Human Dev.

[65] Hammen C (1991). Generation of stress in the course of unipolar depression. J Abnorm Psychol.

[66] Conway CC, Hammen C, Brennan PA (2012). Expanding stress generation theory: test of a transdiagnostic model. J Abnorm Psychol.

[67] Zhand N, Matheson K, Courtney D (2016). Self-harm in child and adolescent psychiatric inpatients: A retrospective study. J Can Acad Child Adolesc Psychiatry.

[68] Kipoulas E, Berzengi A, Kyriakopoulos M (2021). Prevalence and clinical correlates of self-harm and suicidality during admission of children in a mental health inpatient unit. Eur Psychiatry.

[69] Prinstein MJ, Boergers J, Spirito A, Little TD, Grapentine WL (2000). Peer functioning, family dysfunction, and psychological symptoms in a risk factor model for adolescent inpatients’ suicidal ideation severity. J Clin Child Psychol.

[70] Brown RC, Heines S, Witt A, Braehler E, Fegert JM, Harsch D, Plener PL (2018). The impact of child maltreatment on non-suicidal self-injury: Data from a representative sample of the general population. BMC Psychiatry.

[71] Wiens K, Bhattarai A, Pedram P, Dores A, Williams J, Bulloch A (2020). A growing need for youth mental health services in canada: Examining trends in youth mental health from 2011 to 2018. Epidemiol Psychiatr Sci.

[72] Gardner W, Pajer K, Cloutier P, Zemek R, Currie L, Hatcher S (2019). Changing rates of self-harm and mental disorders by sex in youths presenting to Ontario emergency departments: Repeated cross-sectional study. Can J Psychiatry.

[73] Chung D, Hadzi-Pavlovic D, Wang M, Swaraj S, Olfson M, Large M (2019). Meta-analysis of suicide rates in the first week and the first month after psychiatric hospitalisation. BMJ Open.

[74] National Action Alliance for Suicide Prevention (2019). Best practices in care transitions for individuals with suicide risk: Inpatient care to outpatient care.

[75] Edelsohn GA, Braitman LE, Rabinovich H, Sheves P, Melendez A (2003). Predictors of urgency in a pediatric psychiatric emergency service. J Am Acad Child Adolesc Psychiatry.

[76] Rhodes AE, Boyle MH, Bridge JA, Sinyor M, Links PS, Tonmyr L (2014). Antecedents and sex/gender differences in youth suicidal behavior. World J Psychiatry.

[77] Canetto S, Sakinofsky I (1998). The gender paradox in suicide. Suicide Life Threat Behav.

[78] Boyd A, Van de Velde S, Vilagut G, de Graaf R, O’Neill S, Florescu S (2015). Gender differences in mental disorders and suicidality in Europe: results from a large cross-sectional population-based study. J Affect Disord.

[79] Posserud MB, Lundervold AJ(2013) Mental health services use predicted by number of metal health problems and gender in a total propulsion study.The Scientific World Journal247283 10.1155/2013/247283 PMC3654282 23690740

[80] Barbic SP, Leon A, Manion I, Irving S, Zivanovic R, Jenkins E (2019). Understanding the mental health and recovery needs of Canadian youth with mental health disorders: A Strategy for PatientOriented Research (SPOR) collaboration protocol. Int J Ment Health Syst.

[81] Chen A, Dinyarian C, Inglis F, Chiasson C, Cleverley K (2020). Discharge interventions from inpatient child and adolescent mental health care: A scoping review. Eur Child Adolesc Psychiatry.

[82] Sunderji N, de Bibiana JT, Stergiopoulos V (2015). Urgent psychiatric services: A scoping review. Can J Psychiatry.

[83] Zeshan M, Waqas A, Naveed S, Ghulam H, Manocha P (2018). Factors predicting length of stay in an adolescent psychiatric unit, South Bronx, NY: A short report. Can J Psychiatry.

[84] Ninan A, Kriter G, Steele M, Baker L, Boniferro J, Crotogino J (2014). Developing a clinical framework for children/youth residential treatment. Residential Treat Child Youth.

[85] Kinchin I, Tsey K, Heyeres M, Cadet-James Y (2016). Systematic review of youth mental health service integration research. Aust J Prim Health.

[86] Stewart SL, Celebre A, Semovski V, Hirdes JP, Vadeboncoeur C, Poss JW (2022). The interRAI Child and Youth Suite of Mental Health Assessment Instruments: An integrated approach to mental health service delivery. Front Psychiatry.

[87] The Child and Youth Mental Health Lead Agency Consortium (2019) *Realizing the potential: strengthening the Ontario mental health system for children, youth and their families*. https://thefamilyhelpnetwork.ca/wpcontent/uploads/2019/10/Realizing-the-Potential-2018-2019-Provincial-Priorities-Report-10.28.19.pdf;

[88] Smith DH, Hadorn DC (2002). Lining up for children’s mental health services: A tool for prioritizing waiting lists. J Am Acad Child Adolesc Psychiatry.

[89] Stewart SL, Theall LA, Morris JN, Berg K, Björkgren M, Declercq A (2015). interRAI Child and Youth Mental Health Collaborative Action Plans (CAPs) for use with the interRAI Child and Youth Mental Health (ChYMH) Assessment Instrument. Version 9.3, Standard Edition.

[90] Stewart SL, Hirdes JP, McKnight M, Curtin-Telegdi N, Perlman CM, MacLeod K (2017). interRAI child and youth mental health screener (ChYMH-S) assessment form and user’s manual. Version 9.3.

[91] Sundermann JM, DePrince AP (2015). Maltreatment characteristics and emotion regulation (ER) difficulties as predictors of mental health symptoms: Results from a community-recruited sample of female adolescents. J Fam Violence.

[92] Boak A, Hamilton HA, Adlaf EM, Henderson JL, Mann RE (2016). The mental health and well-being of Ontario students, 1991–2015: OSDUHS highlights (CAMH Research Document Series No. 44).

